# Current Understanding of Equine Gut Dysbiosis and Microbiota Manipulation Techniques: Comparison with Current Knowledge in Other Species

**DOI:** 10.3390/ani14050758

**Published:** 2024-02-28

**Authors:** Laurie Boucher, Laurence Leduc, Mathilde Leclère, Marcio Carvalho Costa

**Affiliations:** 1 Department of Veterinary Biomedical Sciences, Université de Montréal, Saint-Hyacinthe, QC J2S 2M2, Canada; 2 Department of Clinical Sciences, Université de Montréal, Saint-Hyacinthe, QC J2S 2M2, Canada

**Keywords:** horse, microbiome, prebiotics, probiotics, fecal microbiota transplant

## Abstract

**Simple Summary:**

Research on equine gut microbiota has grown and gained significant interest in the last decade. Abnormal alterations in the composition of the gut microbiota are called dysbiosis and have been linked to various gastrointestinal tract diseases and remote organs in human medicine, such as the brain and the lung. Strategies to restore the gut microbiota to prevent and treat such diseases are currently being investigated. This review focuses on the current knowledge regarding gut microbiota dysbiosis and microbiota manipulation techniques in horses.

**Abstract:**

Understanding the importance of intestinal microbiota in horses and the factors influencing its composition have been the focus of many studies over the past few years. Factors such as age, diet, antibiotic administration, and geographic location can affect the gut microbiota. The intra- and inter-individual variability of fecal microbiota in horses complicates its interpretation and has hindered the establishment of a clear definition for dysbiosis. Although a definitive causal relationship between gut dysbiosis in horses and diseases has not been clearly identified, recent research suggests that dysbiosis may play a role in the pathogenesis of various conditions, such as colitis and asthma. Prebiotics, probiotics, and fecal microbiota transplantation to modulate the horse’s gastrointestinal tract may eventually be considered a valuable tool for preventing or treating diseases, such as antibiotic-induced colitis. This article aims to summarize the current knowledge on the importance of intestinal microbiota in horses and factors influencing its composition, and also to review the published literature on methods for detecting dysbiosis while discussing the efficacy of gut microbiota manipulation in horses.

## 1. Introduction

Most microorganisms from the human microbial community are found in the gut, comprising over 100 times more genes than the whole human genome [1]. The gut microbiota refers to the collection of microorganisms colonizing the gastrointestinal tract and includes thousands of distinct bacterial species as well as archaea, viruses, protozoa, and fungi [2,3]. This complex polymicrobial community is often called the “forgotten organ” owing to its systemic integration and essential contribution to the host’s health [2,4,5]. The establishment of definitive criteria for what constitutes a “healthy” microbiota has been complicated by the gut microbiota’s significant intra- and inter-individual variability. Nevertheless, it is now well-established that a healthy gut microbiota carries out several functions, including enhancing local mucosal immunity, producing short-chain fatty acids (SCFAs) and developing antigen tolerance [6,7,8]. By preventing the colonization of the intestinal tract by other organisms competing for resources, the microbiota is also involved in protecting the host against infections [7].

Since the microbiota plays a vital role in maintaining health, alterations to its composition may also be involved in pathogenesis or, at least, susceptibility to diseases, as suggested by the occurrence of dysbiosis in various conditions in humans, such as asthma, inflammatory bowel diseases, autism, obesity and colorectal cancer [9]. By aiming at restoring gut microbial composition and homeostasis, gut microbiota modulation strategies such as diet changes, prebiotics, probiotics, and fecal microbiota transplant (FMT) are emerging as possible approaches for the prevention and treatment of conditions in which dysbiosis has been identified [10,11,12].

The human microbiome is beginning to be well documented and understood. However, our knowledge of the equine microbiome is not as extensive. Nevertheless, our understanding of the equine gut microbiota has significantly improved, thanks to the recent advancements in next generation sequencing and bioinformatics [13,14,15]. However, the literature regarding gut microbiota dysbiosis and the efficacy of strategies to modulate the microbiome for the prevention and treatment of diseases remains sparse and primarily descriptive. This review aims to update current knowledge on equine dysbiosis and summarize recent findings on microbiota manipulation techniques. We begin by describing the importance of intestinal microbiota in horses, followed by detailing factors influencing its composition (in positive and negative ways) and factors contributing to gut dysbiosis. Lastly, we discuss methods to detect dysbiosis and the efficacy of gut microbiota manipulation techniques in horses.

## 2. Methodology

Publications were selected if they were English-based and provided original research or reviewed literature on gut microbiota in horses, including dysbiosis and gut microbiota manipulation. Studies with human subjects, laboratory animals and ex vivo studies were included if the findings were pertinent to equine medicine. The most recent literature search was conducted on April 20th, 2023. The following keywords were used to perform most of the literature searches across different databases (PubMed, ScienceDirect, Scopus): equine intestinal microbiota, equine gut microbiome, gut dysbiosis in horses, fecal microbiota transplant in horses, probiotics in horses, gut microbiota manipulation and factors influencing gut microbiota in horses.

## 3. The Importance of Intestinal Microbiota in Horses

A horse’s digestive system is close to 30 meters long and can hold up to 150 liters of organic matter [16]. Over 1000 bacterial species have been identified in the equine gastrointestinal tract [17], and many remain to be discovered. [14,18] The colon and cecum of the horse act as anaerobic chambers, allowing the growth of bacteria considered beneficial to the host. Under oxygen-free conditions, fibrolytic bacteria ferment structural carbohydrates like cellulose into SCFAs such as butyrate, propionate and acetate [16,19,20]. This fermentation allows the horse to obtain substantial amounts of its daily energy requirements from its diet. Given the size of the equine gastrointestinal tract, the large bacterial communities it harbors and its role in the fermentation of the diet, the need to better understand and characterize the intestinal microbiota of horses is undeniable.

**Figure 1:**
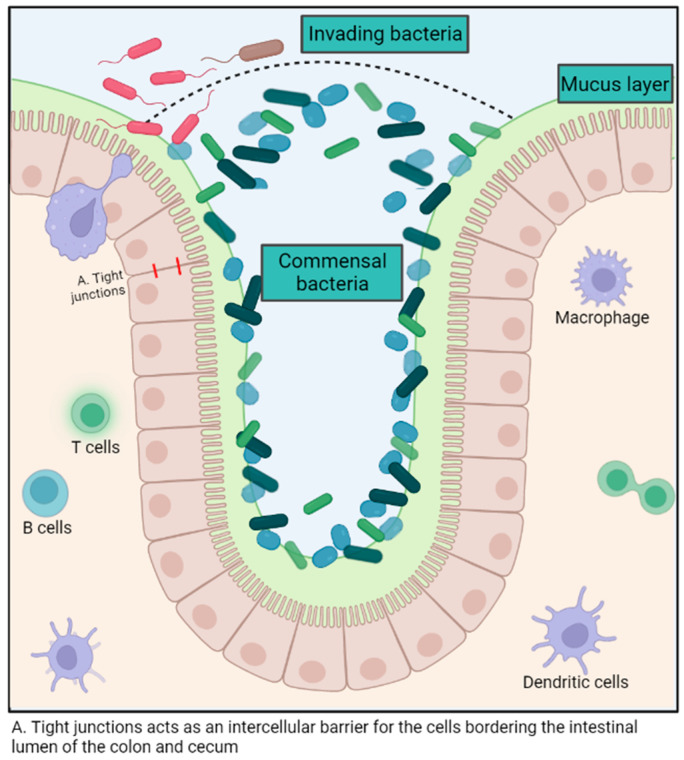
Summary of the role of the gut microbiota in host immunity. Created with BioRender.com.

The intestinal microbiota exerts various immune-modulatory functions. This polymicrobial community aids in preventing the colonization of pathogens in the gastrointestinal tract by competing for the ecological niche and its resources, as well as promoting the production of antimicrobial molecules by intestinal epithelial cells [7,21]. (Figure 1) For example, some *E. coli* strains release bacteriocins that impede the growth of similar species and, therefore, hinder the ability of enterohemorrhagic *E. coli* to proliferate [22]. The intestinal microbiota also enhances local mucosal immunity by stimulating the production of mucus, promoting the induction of B cell and T cell responses against pathogenic microorganisms and improving the function of dendritic cells both systemically and locally [21] (Figure 1). The intestinal mucosa contains nearly 60% of the host’s immunoglobulins and represents a colossal reservoir of immunocompetent cells [7].

The microbiota also supports the regeneration of intestinal mucosal tissues through crypt cell turnover and modulates the permeability of the intestinal epithelium to maximize nutrient absorption [23,24]. For example, the cell turnover in the intestinal crypts and the total intestinal surface area was significantly lower in germ-free mice compared to wild-type mice, thus demonstrating the importance of the microbiota in maintaining intestinal health [25]. Although there is no consensus about the ideal microbiota composition, butyrate producers have been associated with good intestinal health, probably because of the beneficial impact of the metabolite in the mucosal cells. The metabolite can be used as an energy source and maintain intestinal epithelium integrity, notably by reinforcing tight junctions [23,26].

The role of the intestinal microbiota in horse health is just in its infancy, and it might vary from general health [27] to social and behavioral trends [28,29] and performance enhancement [30]. Mechanistic studies in the future will likely reveal more details and guide microbiota manipulation.

## 4. Factors Influencing Microbiota Composition

### 4.1. Diet

Diet is a fundamental factor to consider when assessing gut microbiota, and the detailed impact of diet on a horse’s microbiome has been described elsewhere [31,32,33]. The effect of weaning is discussed later in this review. Abrupt changes in diet can lead to disruptions in the stability of gastrointestinal commensal bacterial species [34,35,36]. Daly et al. showed a lower relative abundance of *Fibrobacter* and Ruminococcaceae in horses fed a concentrate-based diet as opposed to horses fed a forage-based diet [37]. A higher relative abundance of Lachnospiraceae, Bacteroidetes, and the Bacillus-Lactobacillus-Streptococcus group was also noted. These findings agree with the results of other studies [38,39].

The increase in lactic acid-producing bacteria, such as *Streptococcus* spp., can decrease the colon pH through starch fermentation and lead to intestinal acidosis. The lower pH allows other lactic acid-producing bacteria to proliferate, perpetuating dysbiosis [40]. Conversely, *Fibrobacter* spp. and members of the Ruminococcaceae and Lachnospiraceae families are essential to maintaining gut homeostasis by fermenting fibers and producing SCFAs, which have anti-inflammatory and immune-modulating properties. Fiber-based diets are therefore considered optimal for horses, as they aid in maintaining stable bacterial communities and reduce the abundance of lactic acid-producing bacteria, thereby reducing the risk of dysbiosis.

Diet may have an impact beyond the gastrointestinal tract. The so-called “gut–brain axis” has been consistently demonstrated in humans and laboratory animals, but this field of research is in its infancy in horses. A starch-based diet has been suggested to alter the behavior and reactivity of horses in two studies [29,41]. The authors observed that a high-fiber diet was associated with more time dedicated to “investigating” their environment, while a high-starch diet was associated with greater “pace-change” behavior. In addition, the fecal microbial profile of the ponies on the high-fiber diet was associated with calmer and more settled behaviors. Gut microbiota can influence appetite and satiety in humans, and significant differences in behaviors were observed in mice with dietary-induced dysbiosis [42,43,44]. In horses, it is difficult to differentiate the relative contribution of the ingested volume, the time spent masticating, or the effect of simple sugars absorbed from the small intestine versus the microbiota on behavior, and these studies have not fully elucidated this aspect.

### 4.2. Supplements

Various supplements claiming to improve gut health are available. The effects of prebiotics and probiotics on equine gut microbiomes are discussed later in this review. The influence of enzyme supplementation, such as amylase, on gut microbiota remains to be clearly determined. There was no difference in richness (number of different species) after six weeks of amylase supplementation in a study by Proudman et al. [45]. Another study investigating amylase-rich malt extract supplementation observed an increase in the relative abundance of lactate-utilizing bacteria [46]. While the population studied was homogenous and the diet was controlled, the study did not have a control group or crossover design.

### 4.3. Age

The intestinal microbiota changes and evolves during a horse’s life. The gastrointestinal tract is colonized at birth when a dynamic relationship between the host and their microbiota develops [47,48]. Contact with the maternal vaginal microbiota during birth influences intestinal colonization of the foal, but the fecal bacteria of the mare, as well as environmental bacteria, are also important for this initial colonization [49]. Up to weaning, the foal’s gut microbiota composition is influenced mainly by the diet, including the ingestion of colostrum and milk, but also by contact with the udder, skin, maternal hair, and the dam’s feces [32,50]. This is especially important considering the coprophagic behavior of foals during early ages. The dominant bacteria found in foal feces resemble those of adult horses after approximately 60 days of life [13,47,48,51,52]. Still, the overall composition, including the less abundant members of the microbiota, remains significantly different until yearling [48]. Weaning may also considerably affect the gut microbiota composition, which may be attributed to stress. Mach et al. reported a decrease in relative abundances of the genera *Clostridium XIVa*, *Ruminococcus*, *Treponema* and *Fibrobacter*, as well as an increase in *Prevotella*, *Streptococcus* and *Lactobacillus* spp. after weaning at eight months of age [53]. However, another study reported no major change in gut microbiota composition following weaning between 8.5 and 9.5 months old [52], so conclusions on the impact of weaning cannot be drawn. Nonetheless, the most notable changes in microbial composition occur at around 1–2 months of age, which coincides with the ingestion of larger amounts of solid feed [32], as well as the physiological changes observed in other mammalian species.

The bacterial communities in adult (5–12 years) and aged (19 years and older) horses were found to be similar in one study [54]. Still, a reduction in bacterial diversity with age has been reported in horses, similar to changes observed in humans [54,55]. Deterioration of dentition, increased intestinal transit time, changes in diet and decreased energy requirements may partly explain the reduced diversity in the older population, although this remains purely speculative.

### 4.4. Climate, Location and Social Interactions

Seasonal variations and climate can influence the establishment and composition of the gut microbiome, as evidenced by a study by Salem et al. where gut microbiota in horses differed throughout the year without major changes to their management [56]. Although diet and environment clearly impact intestinal microbiota, some microbial changes may be partly explained by food sharing and social interactions [32]. A study investigating semi-feral Welsh ponies found that, while individual variability accounted for 52.6% of the variations in the gut microbiota, group interactions and social behavior also contributed to the overall variations [57]. Interestingly, mother–offspring and stallion–mare interactions lead to more microbiome similarities in that study, suggesting that their microbiome composition may have been influenced by one another. The fecal microbiome of horses differed based on geographic location and habitat, similar to what had been shown in other farm animals [58]. Similar results were obtained in another study, showing that many shared species in the fecal microbiome of healthy horses originated from the same region. Still, other factors, including occupation and diet, were not controlled [59]. Ayoub et al. found significant differences in the fecal microbiota of healthy horses housed in different facilities (teaching horses vs. client-owned), demonstrating the impact of the management and environment on their fecal microbiota [60]. Finally, the higher similarity in the skin microbiota of horses living on the same farm reinforces the conclusion that the environment is an important contributor to the seeding of bacterial species in horses [61].

### 4.5. Exercise

Exercise has been shown to enhance food digestibility in horses [62], but its effect on gut microbiota has just started to be investigated. Changes in gut microbiota composition were induced in horses undergoing an intense 42-day training plan [63]. Interestingly, there was a notable resemblance between the samples collected before and after the training, suggesting an adaptation of the gut microbiota to the exercise program. Therefore, the microbiota may not be altered by exercise per se, but rather by the stress caused by modifications in routine. Conversely, strenuous exercise during a 12-week training period did not affect the fecal microbiome of standardbred racehorses [64].

The systemic consequences of extreme exercise have been demonstrated in endurance horses, who presented different metabolic patterns and expressions of specific genes related to performance [65,66]. However, one study found no association between the gut microbiota of horses competing in an endurance race and blood biochemical and metabolic markers [30]. Studies with controlled diets and larger sample sizes are required before solid conclusions regarding the association between commensal gut bacteria and exercise can be drawn. Improving athletic performance by modulation of the gastrointestinal microbiota has been suggested in laboratory animals receiving a bacterial species (i.e., *Veillonella*) that could metabolize lactate produced during exercise into pyruvate [67].

### 4.6. Transportation

The effect of transportation on gut microbiota has not been extensively investigated, but studies have shown a decline in specific bacterial taxa following transport. Schoster et al. showed a decreased abundance of Clostridia and the order Rickettsiales 12 h following 1-hour transportation [68]. Other studies found a decrease in Streptococci and Bacteroidetes after transportation [69,70], as well as a reduction in different species (alpha-diversity) compared to baseline [69]. However, reduced alpha diversity was also observed in the control group, which could be attributed to stress caused by separation from the transported group. Considering the emotional stress transport can cause in horses, the gut–brain axis could be suggested to explain the microbiota changes observed following transportation [28]. As transportation can be associated with a change in temperature, humidity, abrupt change in diet, and stress, as mentioned earlier, authors investigating the effect of transport on microbiota should select a study design that limits confounding factors and controls for variations within the study population [32].

### 4.7. Antibiotics and Other Medications

Administration of antibiotics may have the most significant effect among all variables altering the microbiome [71] and can lead to antibiotic-induced colitis in humans and horses [72,73]. The disturbance of the local commensal bacterial community (dysbiosis) caused by the administration of antibiotics allows for the colonization or overgrowth of pathogens, such as *Clostridioides* (formerly *Clostridium) difficile* [73,74]. It has been shown that different drug principles significantly impact the microbiota composition in different ways, suggesting that each drug can target specific groups of bacteria (i.e., trimethoprim sulfadiazine (TMS), procaine penicillin and ceftiofur) [75,76,77]. Anecdotally, antibiotics causing antibiotic-induced colitis seem to differ based on geographic locations, highlighting the impact of the environment on gut microbiota.

There is also a consensus that antibiotics are associated with decreased richness and diversity, similar to the effects observed in other species [14], which is believed to be detrimental to health. A rich and diverse microbiota is generally more resilient, meaning that it has a higher capacity to return to its baseline state after perturbation (e.g., after the use of antibiotics). It is also thought that a diverse microbiota contains multiple species with redundant roles in the environment, such as the degradation of bile acids or butyrate production. Thus, if one of those species is affected, the overall consequences to the environment should not be grave.

Although most intestinal microbial alterations caused by antibiotic administration were considered minimal 30 days after treatment in horses in two studies, up to 30% of bacterial taxa remained affected six months following exposure to antibiotics in humans [75,78,79]. Likewise, treatment with metronidazole had long-term consequences in many dogs, including decreased abundances of key beneficial species that have important roles in maintaining intestinal health (including *Clostridium* spp.) [80], which might also be of importance for horses. These results raise serious concerns regarding antibiotics’ long-lasting effects on the gut microbiota. In one study performed in horses, one animal treated with TMS remained in a dysbiotic state, highlighting the interindividual difference in the resilience of their microbiota and, therefore, their capacity to return to a baseline composition [81]. Although the consequences of carrying a subclinical dysbiosis remain uncertain, future approaches considering individual/personalized medicine might use that information.

It should be mentioned that, while antibiotics are detrimental to the intestinal microbiota of horses, their use is unavoidable in many situations, such as post-operatory periods and certain cases of intestinal diseases (i.e., *C. difficile* infection) [82], or in colitis patients presenting neutropenia or signs of sepsis.

Antibiotic administration can affect other organs and have unexpected consequences, especially when given during microbiota development. For example, in humans, asthma and obesity have been associated with antibiotic exposure early in life, and one of the mechanisms could be alterations of the gut (and potentially other organs) microbiota [83,84,85].

Other medications frequently administered may also impact the intestinal microbiota of horses. A transient dysbiosis was observed in the feces of healthy horses following administration of nonsteroidal anti-inflammatory drugs (phenylbutazone and firocoxib), characterized by a decrease in Firmicutes, Ruminococcaceae, Clostridiaceae and Lachnospiraceae [86]. Premedication and general anesthesia affected bacterial community and structure in one study, but it did not affect alpha diversity, and most changes in the microbiota could be attributed to transport and fasting [68]. The studies investigating the impact of helminths and anthelmintics on gut microbiota yield conflicting results and may suggest their effects are minor. Moxidectin and praziquantel decreased alpha but not beta diversity, while fenbendazole, in combination with ivermectin, caused a shift in critical taxa by reducing the relative abundance of Bacteroidetes and increasing the abundance of Firmicutes [87,88].

### 4.8. Diseases

Many diseases have been associated with alterations in the gut microbiota, including diarrhea, colitis, equine metabolic syndrome and colic [89,90,91,92]. It remains unclear if the composition and diversity of the intestinal microbiota influence the susceptibility to developing specific diseases.

Several studies have explored microbial alterations in horses with diarrhea/colitis [59,72,89,93,94,95,96], generally reporting decreased diversity and altered bacterial composition. Decreased abundances in bacteria of the phylum Firmicutes have been reported, and Lactobacillus was more abundant in healthy horses in the study by Zakia et al. [90], whereas other studies reported increased abundance in diarrheic horses [89,96]. These discrepancies across studies may reflect the lack of absolute quantification inherent to amplicon sequencing technologies. Indeed, a greater relative abundance observed during DNA sequencing does not indicate the actual bacterial load in that horse. For example, a horse with diarrhea may have 50% *Lactobacillus* (high abundance), but a healthy horse may have 0.2%. In reality, the healthy horse may have many more *Lactobacillus* in absolute numbers, but it will have a lower abundance because it has many more other bacteria that make up 100% of the total. Likewise, horses that were euthanized because of the severity of colitis had increased abundances of Enterobacteriaceae, *Pseudomonas, Streptococcus*, and *Enterococcus* [93]. Still, it doesn’t mean that the absolute counting of those taxa was increased. Therefore, quantitative studies are necessary to determine if there has been an increase in those groups or if there has been a decrease in the other bacteria. The substantial microbial alterations observed in these studies suggest that dysbiosis may contribute to the pathogenesis or perpetuation of the disease. For instance, one study found that fecal microbiota transplantation was associated with faster diarrhea recovery in puppies with parvovirus infection [97], highlighting the importance of breaking the cycle of dysbiosis to restore a healthy gut environment.

*Salmonella* spp., *Neorickettsia risticii*, *C. difficile* and Equine Coronavirus (ECoV) remain the most tested pathogens in the diagnosis of Equine colitis [98]; however, most cases remain undiagnosed (undifferentiated colitis). Unconventional Clostridia species have been suggested to cause diarrhea in horses [99]. *Clostridium* sensu stricto was increased three-fold in the cecal content of horses with diarrhea compared to healthy horses [90]. Still, another study found no significant difference in the prevalence of *Clostridium innocuum* [100].

Intestinal dysbiosis may also be associated with diseases in remote organs, such as the lungs and feet, as the adaptation of the gut microbiota to environmental changes differed between horses with and without asthma, and fecal samples from horses with chronic laminitis had more species when compared to healthy counterparts [101,102]. Horses with metabolic syndrome have been shown to harbor distinct intestinal microbiota and metabolites compared to healthy controls [92,103]. Furthermore, an in vitro study showed that macrophage cells exposed to fecal extracts obtained from obese horses induced a higher production of proinflammatory cytokines compared to extracts from non-obese horses, suggesting a potential role of the microbiota composition in obese horses [104]. The diseases associated with altered intestinal microbiota have been reviewed elsewhere [13,32].

The current knowledge of factors capable of altering the equine microbiota is summarized in Table 1.

## 5. Detecting Dysbiosis

Currently, no diagnostic methods allow a rapid assessment of the bacterial composition of the horse’s intestinal microbiota, and so the “optimal” or “healthy” gut microbiome composition in horses has yet to be defined. This is partly due to the important intra- and inter-horse variability, and many elements can influence gut microbiota. It is, however, possible to highlight certain phyla and bacterial families that should be present in a healthy equine microbiota of the colon and cecum. When looking at the relative abundance of bacterial communities, Bacteroidetes and Firmicutes are the phyla with the highest abundance in most studies, followed by Proteobacteria and Verrucomicrobia [54,89,90,93,94,95]. Their order of importance tends to vary from one study to another, but current research on horse microbiomes concludes that all those phyla are an important factor for the gut’s health [16,54,89,90,91,93,94,95,101]. However, it is important to note that an overabundance of Proteobacteria has been associated with gut disease [16], so, although they are present in healthy horses, their relative abundance should not be over-represented compared to other phyla.

Interestingly, the family Lachnospiraceae has constantly been associated with healthy horses and other species [54,89,93,96,106,107]. It is suggested that those bacteria produce butyrate by fermentation, which has a protective function for colonocytes [108,109]. Therefore, the absence or decrease in these bacteria could influence the intestinal symbiosis. The Ruminococcaceae family and the genus *Fibrobacter* also play an essential role in maintaining intestinal homeostasis [17,93,96]. As discussed earlier in this review, those bacteria act by degrading the plant wall of the fiber ingested [110]. The presence of these fibrolytic bacteria in the intestinal microbiota is, therefore, essential for digestion in a horse’s diet. Depending on the study, other bacterial families or genera may be found in the microbiota of healthy horses. However, these other bacterial taxa are often variable from one equine to another. The low overall diversity of this microbiota could hypothetically be a consequence of domestication of this species and could be a cause of the increased susceptibility of horses to gut diseases [111]. The concept of a “normal” microbiota should be interpreted according to the microbial communities of similar populations within a comparable environment and signalment rather than as a one-size-fits-all. Furthermore, most studies use fecal microbiota as a marker of the “gut” microbiota. This is inevitable, as feces are the only readily available samples that can be used on a large scale, especially for potential monitoring and diagnostic purposes.

If the criteria to consider a gut microbiota “healthy” seem far from unequivocal, the definition of dysbiosis is even more ambiguous. The bidirectional association between gut dysbiosis and disease is challenging in regard to establishing multifactorial and complex pathologies. Clinical manifestations such as diarrhea, ileus, intestinal hypo- or hypermotility and bloating may occur concomitantly with dysbiosis; however, these signs are mostly non-specific and could be attributable to almost any gastrointestinal disease. A change in an individual’s gut microbiota without substantial modification of its environment or diet may indicate abnormal gut physiology, making microbiota monitoring a potential avenue for the early diagnosis of intestinal diseases [92].

Identifying critical species in the intestinal microbiota that are consistently altered in horses with intestinal diseases might be useful as a proxy for dysbiosis. Decreased richness and diversity are typically present [112], as are other alterations, such as increased Fusobacteria [90] and Clostridia [89], and decreased Lachnospiraceae and Ruminococcaceae. In a study by Arroyo et al., *Escherichia* spp. (Proteobacteria) and *Fusobacterium* spp. were strongly associated with diarrhea [96]. The association between Proteobacteria and dysbiosis has also been demonstrated in other species [113,114,115,116]. The Firmicutes–Bacteroidetes ratio is often presented in microbiota studies and is sometimes used as a marker for dysbiosis in human medicine. Firmicutes and Bacteroidetes represent most of the gut microbial community in horses [13]. The relationship between those two dominant phyla has been associated with dysbiosis in humans, as well as with intestinal diseases in horses [117,118]. However, other studies failed to demonstrate the relevance of this marker for gut dysbiosis [119,120]. This could be explained by the fact that the relative abundance of Firmicutes and Bacteroidetes is highly variable between individuals and can be influenced by lifestyle, diet, exercise, and antibiotic administration. It is, therefore, difficult to reach a conclusion on the accuracy and usefulness of the Firmicutes/Bacteroidetes ratio as a marker of gut dysbiosis in horses. The Firmicutes/Proteobacteria ratio was associated with post-partum colic in mares, but its value as a marker of gut dysbiosis and as a predictor of colic on a broader population remains undetermined [91].

A qPCR-based test, called the dysbiosis index, has been developed to indicate dysbiosis in dogs and cats without the need for a whole microbiota analysis [80,121,122]. The test measures seven different bacteria in addition to the total number of bacteria, some typically seen in health (e.g., *Clostridium hiranonis*) and some generally associated with inflammation (e.g., *Escherichia coli*). Furthermore, this method allows for absolute quantification of each taxon, establishing reference values for the actual amount of those bacteria in feces. Similar studies investigating this in horses have yet to be performed.

## 6. Techniques of Microbiota Manipulation

**Figure 2:**
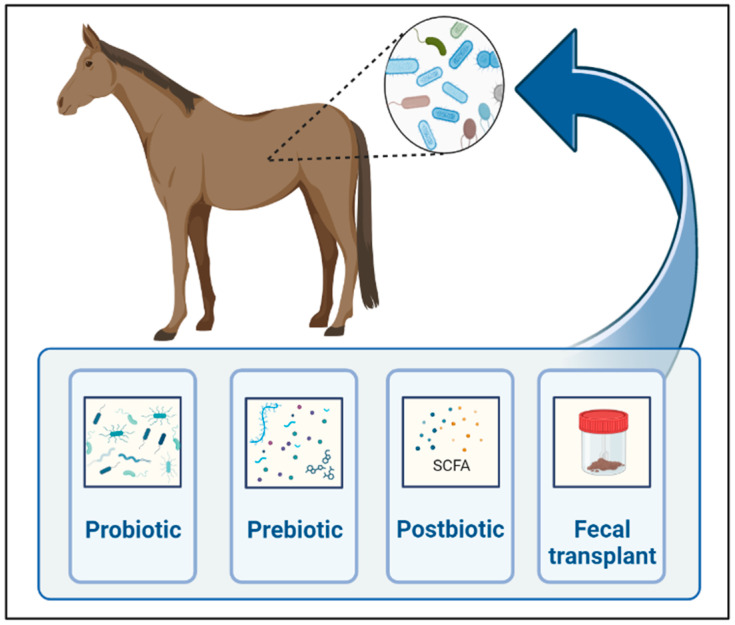
Main techniques currently used for gut microbial manipulations in horses. Created with BioRender.com.

As we learn more about the gut microbiome’s influence on gastrointestinal and remote organ diseases in horses, the modulation of gut microbiota to restore microbial homeostasis gains interest in regard to the prevention and treatment of various conditions. Strategies to modulate gut microbiota include prebiotics, probiotics, symbiotics, postbiotics, antibiotics, bacteriophages, fecal microbiota transplant, and diet (Figure 2).

### 6.1. Diet

Diet plays a crucial role in gut microbiota and can be used to modulate its composition. Differences between humans fed a high-fiber diet and a standard Western diet are marked, and changes in gut microbiota composition and diversity have been shown in individuals with variable diets. This can be explained by the fact that specific diets can promote the proliferation of certain bacteria and modulate the production of metabolites, consequently modifying intestinal pH and other vital factors in the microbial balance [123,124,125]. The applicability of diet for modulating gastrointestinal microbiota in clinical situations is limited by the anorexia frequently observed in severe disease, as well as the fact that changes in microbial communities can take days or weeks to occur.

### 6.2. Probiotics

Probiotics are living microorganisms with the ability to confer benefits to the host. Common examples include *Saccharomyces cerevisiae*, *Lactobacillus*, *Bifidobacterium* and *Enterococcus* spp. Probiotic strains can be administered to prevent colonization by pathogens [126], also utilizing their immune-modulatory capacity to suppress inflammation and improve gut barrier function by upregulating tight junction proteins [10,127].

Some limitations to the use of probiotics are inferred, and results from the literature on horses and other species remain conflicting. To exert their beneficial effects, probiotics must survive the acidic conditions of the stomach and undertake the difficult task of colonizing the gut [128], which is notably more challenging in healthy individuals bearing a well-established and resilient microbiota. The peculiar anatomy and physiology of the equine digestive system may present even more obstacles (e.g., cecal fermentation) in terms of the success of probiotics remaining viable when achieving the distal gut. Finally, regulatory legislations forbid using alternative microbial strains which are sometimes more adapted to that specific environment (i.e., commensal clostridial species).

A recent review has highlighted the inconsistency and disagreement among studies investigating the impact of probiotics in horses, highlighting the need for well-controlled studies using larger sample sizes [129].

Current studies’ scarcity of consistent and significant benefits of probiotics in gut microbiota may reflect a genuine lack of efficacy or inadequate statistical power to detect benefits. One of the potential explanations for the inconsistent results of different studies could lie in the content of viable microorganisms in the probiotics used. The microbial content of eleven bacteria and yeast-based probiotics marketed for use in horses were evaluated by Berrata et al., and none met their label claim based on culture-dependent and independent techniques [130]. For example, *S. cerevisiae* was cultured from only one of the four probiotics claiming to contain this yeast. Another study showed that only 4 of 15 bacteria-based probiotics evaluated met their label claim of viable microorganisms [131]. Therefore, the quality of viable yeast and bacteria in the probiotics currently marketed for animals is highly questionable. Randomized controlled trials with larger study populations and standardized administration, including duration, dosage and frequency, are definitely needed.

Probiotics may be helpful in modulating gut microbiota composition in foals, as the period between birth and 60 days of life might be the best opportunity to modulate the dynamically changing intestinal microbiota. However, no major significant differences were found when supplementating *Lactobacillus* and *Bifidobacterium* to neonatal foals [132]. One study demonstrated a decrease in the incidence and duration of diarrhea in foals after administering a probiotic containing *Lactobacillus* and *Bifidobacteria* [133]. Conversely, foals treated with a similar probiotic were more likely to develop diarrhea compared to the control group [134]. Moreover, using *Lactobacillus pentosus* increased the risk of diarrhea and the need for veterinary care in foals [135].

The most studied, and perhaps the most promising, probiotics used in adult horses are *Saccharomyces cerevisiae* and *boulardii*, two species of non-pathogenic yeasts that are closely related and have the potential to produce proteases able to degrade toxins [136]. In addition, they carry important amounts of mannan oligosaccharide, a constituent of their cell wall that might be used by bacteria as substrate, possibly favoring the growth of beneficial species of bacteria in the gut. Supplementation with *Saccharomyces* has been evaluated in horses [70,137], and the strains could be cultured from feces, supporting that the yeasts were viable in horses with colitis [138,139,140]. However, in one study *S. boulardii* was no longer detectable in the feces ten days after cessation of supplementation [140], suggesting that *Saccharomyces* is likely to survive the acidic pH of the stomach in horses but may not colonize the gastrointestinal tract permanently. Some yeast can grow at a pH of three, and some can tolerate and survive in environments with a pH as low as 1.5 [136]. It is counter-intuitive to supplement bacterial probiotics concomitant with the treatment with antimicrobials; therefore, the use of *Saccharomyces* spp. might be a good strategy in those cases [136].

The supplementation of *S. cerevisiae* can increase the relative abundance of cellulolytic bacteria in the feces of horses fed high-concentrate diets [70,138]. It is also thought that the yeast might influence microbiota physiology by increasing the activity of some enzymes related to plant digestion without necessarily changing the proportions of cellulolytic bacteria [138]. Probiotic products containing lactate-utilizing bacteria may also help inhibit the decline in pH that occurs with a high-starch diet [141]. As for the potential benefits of probiotic supplementation in horses fed a high-forage diet, three publications did not report a significant modification of fibrolytic bacteria following supplementation [142,143,144]. In contrast, one reported an increased relative abundance of members of the Lachnospiraceae family, notably *Roseburia*, a butyrate-producing bacteria [145]. Furthermore, fecal diversity was increased in horses supplemented with *S. cerevisiae* regardless of their diet (high-concentrates or high-forage) [137].

In horses with enterocolitis, Desrochers et al. found a significant decrease in the duration of diarrhea in horses receiving *S. boulardii* [140]. However, in a randomized and controlled prospective study using a similar supplement with a larger sample size, supplementation had no significant effect on the clinical outcome (diarrhea, leucopenia, appetite, survival) [139]. Some conflicting results on the effects of *Saccharomyces* suggest that dosage and diet might influence their efficacy [143,146]. A recent review on the use of Saccharomyces in horses has been published [146].

Future perspectives for the use of probiotics in horses include enhancing performance strains and the development of genetically edited organisms. As mentioned in session 4.5, supplementation of laboratory animals with a strain of *Veillonella* increased performance by 13% due to the capacity of the bacterium to metabolize lactate produced during exercise and produce pyruvate [67]. Other important metabolites of intestinal origin have also been identified in high-performance horses [147]; thus, new genetic editing techniques that are currently available provide an unprecedented opportunity to infer the ability of certain bacteria to produce compounds of interest, such as vitamins or VFAs [148].

### 6.3. Prebiotics

Prebiotics are defined by the International Scientific Association for Probiotics and Prebiotics as “substrates that are selectively utilized by host microorganisms conferring a health benefit”, and they are most commonly oligosaccharides, such as fructo-oligosaccharides (FOS) or mannan-oligosaccharides (MOS) [126]; in other words, they are fibers that serve as a food source for beneficial bacteria, typically culminating with the production of SCFAs [10]. Prebiotics can also inhibit colonization by pathogenic strains by interacting with bacterial receptors and competing with attachment sites [149,150]. However, prebiotics are not strictly selective of beneficial species and might depend on the pre-existing microbiota of their subjects. In fact, prebiotics are so powerful that they have been used as a model for induction of equine acute laminitis [151]. The fermentation of the massive amounts of oligofructose by *Streptococcus* spp. causes a marked decrease in pH that kills other non-resistant species, releasing vasoactive molecules (i.e., lipopolysaccharides) that will culminate with the onset of the disease.

Several studies evaluating the impact of prebiotics on the horse-hindgut microbiome have been reported, but most of them either enrolled a low number of horses per group, included many variables, evaluated different products, or were performed in vitro [152,153,154,155,156,157,158,159,160,161,162]. Furthermore, it is not rare for the private companies producing those compounds to sponsor the studies, and therefore results should be interpreted with particular caution as they tend to emphasize the beneficial changes in the microbiome. Other studies have evaluated the impact of prebiotics on the gastric microbiome [163,164].

### 6.4. Other

Symbiotic supplements are a combination of both prebiotics and probiotics to obtain synergism. For example, a symbiotic supplement might contain *E. faecium*, *L. acidophilus* and FOS or MOS.

Postbiotics are defined as metabolites and soluble byproducts with biological activities released by components of the intestinal microbiota. The most well-known postbiotics are SCFAs, which play a role in the integrity of the gut barrier and local immune modulation [8]. SCFAs are produced through the fermentation of fibrolytic bacteria, such as Bacteroidetes, and supply most of the daily energy requirements to the horse. The administration of postbiotics to prevent or treat inflammatory conditions has gained significant interest in human medicine. Notably, supplementing drinking water with propionate or acetate reduced inflammatory infiltration in the airways of mice sensitized to house dust mites in a model of allergic asthma [117]. Microencapsulated butyrate was given to horses with experimentally induced carbohydrate overload, showing only minor benefits, not influencing fecal consistency, intestinal pH, villus length, or feed intake [165].

Antibiotic administration to modulate gut microbiota is controversial as it depletes most of the gut microbiome, including beneficial bacteria, and does not promote judicious use of antibiotics. Although each antibiotic principle will modulate microbiota composition in a specific manner [77], the extent of changes caused by those drugs cannot justify their use to treat dysbiosis.

Bacteriophages are prokaryotic viruses that propagate via lysis of bacteria and, therefore, could affect the gut microbiome and metabolome. Their potential in selectively targeting pathogenic organisms is under investigation in human medicine, but has not been studied in horses [166,167].

### 6.5. Fecal Microbiota Transplantation

FMT refers to the administration of a suspension of feces from a healthy donor into a patient to restore the balance of the gut microbial community. It has become an accepted treatment for recurrent *C. difficile* infection in humans, with up to 95% success rates in the resolution of diarrhea [168,169].

The storage and preparation of fecal samples for FMT may significantly impact the microorganisms’ viability and, consequently, the efficacy of the procedure [170,171]. Guidelines for preparing FMT solutions for horses have been proposed based on experts’ experiences [172]. Briefly, a bucket of feces collected from a healthy donor is diluted in approximately five liters of saline or water, and the solution is strained and frozen. However, scientific evidence demonstrating bacterial viability after the processing of feces is scarce. One study showed that freezing at −20 °C with subsequent exposure to the proximal GI tract enzymes significantly decreased bacterial viability [171]. In another report, freezing decreased the bacterial viability in the feces of 10 healthy horses by almost half [173]. When processed fresh, three fecal samples from one horse showed no major difference in bacterial viability before and after FMT preparation. Pretreatment of the patient with proton-pump inhibitors before FMT has been suggested [172]; however, the time necessary for those medications to significantly increase the gastric pH can be up to 48 h. In addition, administration of buffering solutions (i.e., 1M solution of sodium bicarbonate) might be helpful. It is uncertain how the concomitant use of antimicrobials affects the transplanted bacteria, but treatment with those drugs should be avoided unless otherwise recommended. Therefore, current evidence suggests that FMT solutions should be prepared and administered freshly (without freezing) as soon as possible with minimal exposure to oxygen. One difficulty this recommendation might impose is the availability of a healthy donor from whom feces could be promptly collected, and the criteria for donor selection should be a topic for extensive discussion. Briefly, donors should be tested for the main enteropathogens, including bacterial, viral and parasitic agents. It is currently unknown if the donor’s microbiota composition can influence the engraftment efficiency, as has been suggested in humans [174].

Currently, published data on the efficacy of FMT in horses is conflicting. McKinney et al. found higher diversity and significant changes in the composition of fecal samples of five geriatric horses with diarrhea after three treatments with FMT [94]. However, no controls were included, precluding major conclusions as to whether FMT caused the changes or were inherent to transient diarrhea. Furthermore, two horses did not survive treatment. Later, the same group published another study featuring 22 horses with colitis, of which 12 were treated with FMT for three days [95]. Although treated horses showed a greater diarrhea resolution and faster microbiota recovery, control animals started the study with statistically worse clinical parameters. Furthermore, control horses were sampled in a different hospital, highlighting the difficulty of performing controlled studies using a large sample size in equine medicine.

Contrary to those two reports, several other studies reported no impact of FMT on the fecal microbiota of horses. Kinoshita et al. induced dysbiosis by treating nine horses with metronidazole but failed to find a protective effect between the groups receiving or not receiving FMT [175]. However, only three horses per group were used, and only 500 g of feces were transplanted. Costa et al. also found no effect of FMT on fecal microbiota in six horses with diarrhea throughout seven days. However, no long-term evaluation was performed, and the recipient group was small and heterogenous (age differences, clinical signs and treatment) [106]. Laustsen et al. treated ten horses suffering from Free Fecal Water syndrome (a condition characterized by the presence of water along with well-formed fecal balls) with FMT for five days [176]. No significant changes were found in the microbiota of the treated animals but, again, only 500 g of feces were used to prepare the FMT solution. A decrease in the symptom’s severity was reported, but horses were evaluated by their owners non-blinded, which might have introduced bias to their evaluation. Quattrini et al. performed a retrospective study using records from 111 cases of colitis from two equine hospitals [177]. Non-treated horses had significantly shorter hospitalization times than the group receiving FMT, and there were no statistical differences in time to normalize fecal consistency, clinical parameters, or mortality. It should be mentioned that studies like this are susceptible to bias, as FMT might have been given as a last resource in the treatment of patients not responding to conventional therapy, which could explain the longer time of hospitalization. Once again, the current evidence of the efficiency of FMT in horses supports the urgent need for prospective multicenter case-control studies.

The impact of a protocol using a dose of bacteria six times higher than recommended has been evaluated in horses with antibiotic-induced dysbiosis [81]. In that study, an FMT solution concentrated by centrifugation given twice a day induced a more homogeneous change in microbiota composition compared to both animals receiving the regular FMT and to controls (*n* = three horses per group). However, the changes observed were not towards the baseline composition of their microbiota, and the concentrated FMT solution contained high abundances of *Escherichia* spp., which probably increased during the manipulation of the feces from the donor. Despite the small number of animals used in that study, it suggests that the dose of bacteria might be important for the procedure’s efficacy in horses. However, we are still far from being able to restore the normal microbiota of horses.

It has been shown in human medicine that delivering the FMT solution directly in the large intestine by enema or endoscopy is associated with greater success in colonizing and resolving dysbiosis and clinical diseases [178,179]. This is also the preferred administration route in dogs. However, horses have particularly long and small colons that might preclude the solution administered via enema from travelling to the large colon and cecum, especially with the high peristalsis in animals with diarrhea.

Table 2 summarizes the shreds of evidence of methods used to manipulate the equine microbiota obtained from this review.

Limitations to this review include the use of English-based literature only, as other papers on equine microbiota may have been published in other languages. It is also important to consider the variation in results with different sequencing platforms and DNA databases, which may have differed for the articles cited in this review. As this is a difficult control limitation, it is essential to be aware of it when considering the results of DNA sequencing. More studies using a larger sample size and similar methodologies are necessary before a meta-analysis can be performed.

## 7. Conclusions and Future Directions

This review underlines the lack of data regarding the methods used to diagnose gut dysbiosis in horses and the lack of a definition for what constitutes an “ideal” microbiota. The studies evaluating the efficacy of microbiota manipulation techniques in horses are scarce compared to other species and yield conflicting results. Standard protocol guidelines for FMT should be evaluated by controlled studies to prove the potential efficiency of the procedure. The causal relationship between intestinal microbiota composition and several conditions and diseases in horses also awaits further investigation. Controlled studies with larger sample size populations are needed to determine the efficacy of microbiota manipulation techniques in the equine species. Finally, developing faster and quantitative methods to detect dysbiosis in horses could facilitate research in this field and be used in a clinical setting in the future.

## Figures and Tables

**Table 1:** Summary of findings of studies investigating factors affecting the microbiota of horses.

Factor Influencing the Equine Microbiota	Reference	Key Findings
Diet	Julliand et al., 2017 [31] Garber et al., 2020 [32] Hesta et al., 2020 [33]	Diet alters the composition of the horse’s gut microbiome.
	Muhonen et al., 2008 [34] Muhonen et al., 2009 [35] Fernandes et al., 2014 [36]	The gut microbiome of horses is altered by abrupt changes in diet.
	Daly et al., 2012 [37] Zhu et al., 2021 [38] Warzecha et al., 2017 [39]	Non-structural carbohydrate (concentrate) based diets promote the growth of lactic acid-producing bacteria (e.g., *Bacillus-Lactobacillus-Streptococcus* group). Fiber-based diets are considered optimal for horses because they promote the growth of bacteria associated with good intestinal health (e.g., Ruminococcaceae, Lachnospiraceae, *Fibrobacter*).
	Destrez et al., 2015 [41] Bulmer et al., 2019 [29]	Non-structural carbohydrate-based diets have been suggested to alter equine behavior (e.g., “pace-change” behavior) and reactivity compared to high-fiber diets.
Supplements	Proudman et al., 2014 [45]	Six weeks of amylase supplementation did not change microbiota richness.
	Proudman et al. 2015 [46]	Amylase-rich malt extract increased the relative abundance of lactate-utilizing bacteria.
Age	Husso et al., 2020 [49]	The colonization of the newborn foals originated from the maternal vaginal and fecal microbiota, as well as environmental bacteria.
	Quercia et al., 2019 [50] Garber et al., 2020 [32]	Until weaning, diet (milk) the dam’s udder, skin, hair, and feces influence the foal’s microbiota.
	Lidenberg et al., 2019 [52]	At approximately 60-days-of-age, the dominant bacteria found in the foal feces are similar to those found in adult horses, but the overall composition remains significantly different until yearling.
	Dougal et al., 2014 [54]	Similar bacterial communities were found between adult and aged horses. However, a reduction in bacterial diversity was observed in older animals.
Climate	Salem et al., 2018 [56]	Seasonal differences in the microbiota composition were observed throughout the year.
Social interactions	Antwis et al., 2018 [57]	The composition of the gut microbiota may be influenced by group interactions and social behavior.
Geographic location	Ang et al., 2022 [58] Arnold et al., 2021 [59] Ayoub et al., 2022 [60] Kaiser-Thom et al., 2021 [61]	The fecal and skin microbiota of horses tended to differ based on geographic location and habitat, suggesting that environment and management might influence the microbiota composition.
Exercise	Pagan et al., 1998 [62]	Exercise improved feed digestibility in horses.
	Almeida et al., 2016 [63]	Exercise significantly changed the microbiota of unfit horses. A training program was also associated with specific changes in the gut bacteria.
	Janabi et al., 2017 [64]	No changes were found in the microbiota of standardbred racehorses after 12 weeks of intensive training.
	Plancade et al., 2019 [30]	No associations were found between gut microbiota and blood biochemical and metabolic markers in horses competing in endurance races.
	Górniak et al., 2021 [105]	There was a significant increase in Firmicutes and Bacteroidetes after exercise.
Transportation	Schoster et al., 2016 [68] Perry et al., 2018 [69] Faubladier et al., 2013 [70] Mach et al., 2020 [28]	There is evidence that transportation can decrease diversity as well as the abundance of certain bacterial taxa (e.g., Clostridia, Rickettsiales, Streptococci, Bacteroidetes). Those changes are likely associated with the stress caused by transportation.
Antibiotics and other medications	Barr et al., 2013 [73] Harlow et al., 2013 [74] Di Pietro et al., 2021 [14]	The administration of antibiotics can lead to antibiotic-induced colitis, which allows for the overgrowth of pathogens and leads to a decrease in the richness and diversity of species.
	Costa et al., 2015 [75] Liepman et al., 2022 [76] Gomez et al., 2023 [77]	Each drug principle has a distinct effect on the composition of the intestinal microbiota.
	Costa et al., 2015 [75] Liepman et al., 2015 [78] Di Pietro et al., 2023 [81]	Most of the microbial changes caused by antibiotic administration were considered minimal 30 days after treatment, but some individuals might bear a less resilient microbiota.
	Whitfield-Cargile et al., 2018 [86]	After administration of nonsteroidal anti-inflammatory drugs, transient dysbiosis was observed in the feces of healthy horses.
	Schoster et al., 2016 [68]	Pre-surgery medication and general anesthesia affected the bacterial community. However, most of the changes in the microbiota could be attributed to transport and fasting.
	Kunz et al., 2019 [87] Sirois R., 2013 [88]	Studies on the effects of helminths and anthelmintics on gut microbiota are conflicting.
Diseases	Arnold et al., 2021 [72] Arnold et al., 2021 [59] Zakia et al., 2023 [90] Costa et al., 2012 [89] Ayoub et al., 2022 [93] McKinney et al., 2020 [94] McKinney et al., 2021 [95] Arroyo et al., 2020 [96]	Many diseases have been associated with changes in the gut microbiota, including colitis, equine metabolic syndrome, and colic. In general, decreased diversity and altered bacterial composition have been reported. Many studies show a decrease in the relative abundance of the Firmicutes phyla in diarrheic horses and an increase in other taxa, such as *Lactobacillus*, *Pseudomonas*, *Streptococcus*, *Enterococcus*, and Enterobacteriaceae.
	Uzal et al., 2022 [99] Zakia et al., 2022 [100] Zakia et al., 2023 [90]	Unconventional Clostridia species have been suggested to cause diarrhea in horses, as *Clostridium sensu stricto* was increased three-fold in the cecal content of horses with diarrhea. Another study found no significant difference in the prevalence of *Clostridium innocuum*.

**Table 2:** Summary of findings and conclusion on the main techniques currently used for gut microbial manipulations in horses.

	Findings	Conclusions
Probiotic	There are inconsistencies in the results and conclusions of different studies. Many probiotics marketed for horses do not comply with their labels. Studies have demonstrated the potential of high doses of the yeast *Saccharomyces* to modulate the gut microbiota in horses.	Well-controlled studies with larger sample sizes should be conducted to conclude the actual efficiency of probiotics in horses. Saccharomyces cerevisiae or boulardii are perhaps the most promising probiotics for adult horses.
Prebiotic	Prebiotics (e.g., oligosaccharides) have great potential to modify the hindgut microbiota of horses when given at high doses. Most of the studies included small sample sizes, had many variables, evaluated different products, or were performed in vitro.	It is difficult to conclude from the available data benefits and adequate doses of prebiotics to modulate the equine gut microbiome. Well-controlled studies with larger sample sizes should be performed.
Fecal microbiota transplantation (FMT)	There are inconsistencies in the results and conclusions of different studies. Freezing feces before FMT likely decreases bacterial viability.	Multicenter, well-controlled studies with larger sample sizes should be performed to prove the clinical efficacy of FMT. Standard protocol guidelines for FMT should be based on the results of controlled studies.
